# Shear Bond Strengths between Three Different Yttria-Stabilized Zirconia Dental Materials and Veneering Ceramic and Their Susceptibility to Autoclave Induced Low-Temperature Degradation

**DOI:** 10.1155/2016/9658689

**Published:** 2016-05-12

**Authors:** Manoti Sehgal, Akshay Bhargava, Sharad Gupta, Prateek Gupta

**Affiliations:** ^1^Department of Prosthodontics, Faculty of Dental Sciences, SGT University, Gurgaon, India; ^2^Department of Prosthodontics, Institute of Technology and Sciences, Centre for Dental Studies and Research, Muradnagar, Ghaziabad, India; ^3^Department of Periodontics, PDM Dental College, Bahadurgarh, India

## Abstract

A study was undertaken to evaluate the effect of artificial aging through steam and thermal treatment as influencing the shear bond strength between three different commercially available zirconia core materials, namely, Upcera, Ziecon, and Cercon, layered with VITA VM9 veneering ceramic using Universal Testing Machine. The mode of failure between zirconia and ceramic was further analyzed as adhesive, cohesive, or mixed using stereomicroscope. X-ray diffraction and SEM (scanning electron microscope) analysis were done to estimate the phase transformation (m-phase fraction) and surface grain size of zirconia particles, respectively. The purpose of this study was to simulate the clinical environment by artificial aging through steam and thermal treatment so as the clinical function and nature of the bond between zirconia and veneering material as in a clinical trial of 15 years could be evaluated.

## 1. Introduction

The most recent introduction to the dental ceramics family is zirconia, which in its pure form is a polymorphic material. Fixed partial denture and crowns are available options for replacing lost tooth structure. Fabrication of fixed prosthesis involves layering of metallic substructure or all ceramic core structure with an aesthetic ceramic veneering material. The underlying substructure provides the required strength and the overlying ceramic gives the required esthetics [1].

Yttrium oxide partially stabilized tetragonal zirconia polycrystal (Y-TZP) was introduced in dentistry as a core material in the 1990s. The name of zirconium is said to be derived from the Persian word “Zar”-“gun” meaning golden in color [2].

Many studies on the use of zirconia have been conducted, but some features still remain unclear. There is a lack of information on how temperature change and water treatments affect the material properties and clinical durability of veneered zirconia cores. Unlike other prostheses in the body, oral prosthesis is constantly subjected to moisture with fluctuating pH and temperature. Recent studies have shown an indication that water molecules have an influence on the bond between veneered and veneering material [3]. Water molecules can penetrate the zirconia lattice during exposure in a humid atmosphere. Slow surface transformation to the stable monoclinic phase occurs through environmental stresses, usually in the presence of water molecules, hot water vapor, or body fluids such as saliva [4–7].

The substructure (zirconia core) of the restoration is covered by veneering ceramic. Studies have shown the bond between the zirconium core and the veneering ceramic to be influenced by water molecules which eventually affects the clinical durability of the restoration in function [8, 9].

Aging of zirconia may have detrimental effects on its bonding with veneering ceramics; mechanical stresses and wetness exposure accelerate this process [10]. Presently, different companies are providing milled zirconia cores from presintered zirconia blocks and are offering long time warranty (15 yrs) for their products [11]. On observing the growing trends and future possibilities, it is very important to understand the clinical durability, limitation, and function of the veneered zirconia as a restorative material. This study was undertaken to investigate and compare the bond strengths of commercial zirconia framework materials with veneering material as influenced by (steam and thermal treatment) artificial aging. In this study, SBS testing and fracture surface analysis was carried out to microscopically characterize the failure modes and to evaluate and compare the phase transformation (m-phase fraction) and surface grain size (*μ*m) using X-ray diffractometer and SEM (scanning electron microscope), respectively, between aged and nonaged specimens. The objective of this study was to simulate the clinical environment so the durability, clinical function, and nature of the bond between zirconia and veneering material, as in a clinical trial of 15 years, can be evaluated. The null hypothesis tested was that the bond strength between different zirconia framework and veneering ceramic will not be affected by artificial aging.

## 2. Materials and Method

### 2.1. Preparation of Zirconia Core Specimens

Three types of zirconia were selected for this study: Cercon (DeguDent, Hanau, Germany), Ziecon (Jyoti Ceramic Industries Pvt., Ltd., Nashik, India), and Upcera (Shenzhen Upcera Co., Ltd., China) (Table 1). A total of 90 samples in the form of disc with each zirconia system having 30 samples were fabricated from their respective zirconia blocks of 98 mm diameter × 10 mm height by CAD/CAM procedure. A CAD model of the sample is designed for the purpose of the study and converted into Stereolithographic (STL) format in the computer. The CAD design was used by milling machine and samples of 7 mm diameter × 3 mm height were milled out from the standard blanks of partially sintered zirconia. As per the manufacturer instructions the milled zirconia was sintered (Table 2).

### 2.2. Aging Test (Thermal and Steam Treatment)

Though thermocycling has been advocated in the literature as a conventional aging test, autoclaving induced low-temperature degradation is an established method for accelerated aging of Y-TZP materials. Autoclaving at 134°C for 5 hours is the standard aging protocol according to ISO 13356 valid for Y-TZP implants for surgery. 20 samples from each group were autoclaved at 134°C for 5 hrs to simulate oral conditions for 15 years [3].

### 2.3. Preparation of Core-Veneer Specimens

The prepared samples were steam cleaned and taken up for ceramic layering application. Ninety samples of different zirconia core material were layered using VITA VM9 (VITA Zahnfabrik, Bad Säckingen, Germany) veneering ceramic material (Table 3). First, the liner material, which was a single, thin, continuous layer supplied by the manufacturers, was applied and fired independently according to the manufacturer's instructions (Table 4). The veneering procedure was done using the manual layering technique. For the application of base dentin, dentin powder and modelling liquid were mixed according to the standard procedure and a customized metallic jig was used to achieve a uniform thickness of 1 mm. Similarly application of 1 mm enamel layer was done. Finally, the samples were finished to achieve the uniform thickness of 2 mm. The prepared core-veneer disks were fired in a programmable vacuum porcelain furnace (VITA Vacumat 4000 Premium T, VITA Zahnfabrik, Bad Säckingen, Germany) according to the firing programs provided by the manufacturer (Table 4). A total thickness of each sample including the zirconia substructure of approximately 5 mm was obtained.

### 2.4. Shear Bond Strength (SBS) Test

A metal jig was used for holding the samples to determine the shear bond strength. The samples were held in such a manner that the junction of zirconia substructure/veneering ceramic interface was faced towards the chisel load applicator. Universal Testing Machine (Model 3345, Instron Corp., Norwood, MA, USA) with a 10 kN load cell and crosshead speed of 0.5 mm/min was used. A chisel load applicator was used to direct a parallel shearing force to the substructure/veneer ceramic interface. The fracture load was obtained with the help of the graph on the digital monitor attached to the machine. The drop in the graph determined the point of debonding (fracture load) of each sample. Fracture load (kg) was converted to the shear bond strength (MPa) by use of the cross-sectional area of disc. The following formula was used to calculate the shear bond strength (MPa):

(1) $$\begin{array}{c} \text{Shear bond strength}\left(\;MPa\;\right)=\frac{\text{Fracture load}\left(kg\right)}{\text{Area of disc}\left(mm^{2}\right)}. \end{array}$$

### 2.5. Analysis of Bond Failure

After shear bond strength analysis, all the debonded samples were analysed under a stereomicroscope (20x) to evaluate the nature of bond failure. Each specimen was placed on carbon coated flat platform in such a manner that the surface between zirconia substructure and veneering ceramic faces the pointer of stereomicroscope, so as to identify whether there was cohesive failure, adhesive failure, or a combination of both, that is, mixed failure.

### 2.6. Analysis of Phase Transformation

The crystalline phases on the surfaces of the specimens were analysed by a Philips X'Pert 1 X-ray diffractometer. Scans were performed in the 2Θ range of 25° to 35° with a step size of 0.01° and 0.5 s/step interval. The m-phase fraction (*X*_m_) was calculated by equation given by Garvie and Nicholson. The m-phase fraction (*X*_m_) was calculated by the following equation [12]:

(2) $$\begin{array}{c} X_{m}=\frac{\left[I_{m}\left(111\right)+I_{m}\overset{-}{\left(111\right)}\right]}{\left[I_{m}\left(111\right)+I_{m}\overset{-}{\left(111\right)}+I_{t}\left(111\right)\right]}\times 100, \end{array}$$

where *I*_m_(111) is intensity of the peak (areas under the 31.5° peak) that represents the m-phase, $I_{m}\overset{-}{\left(111\right)}$ is intensity of the peak (areas under the 28.2° peak) that shows the m-phase, and *I*_t_(111) is intensity of the peak (areas under the 30.3° peak) that represents the t-phase.

### 2.7. Evaluation of Surface Particle Size

**Figure 1:**
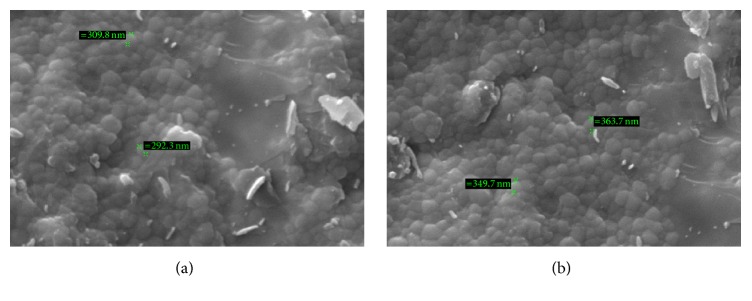
(a) Microstructural analysis of grain size of zirconia specimens without autoclave treatment (without artificial aging) under scanning electron microscope at the magnification of 30,000x. (b) Microstructural analysis of grain size of zirconia specimens with autoclave treatment (with artificial aging) for 5 hours under scanning electron microscope at the magnification of 30,000x.

After phase analysis, to observe the surface microstructure for the surface particle size evaluation, specimens were sectioned and polished with a diamond wheel and grinding machine and coated with gold using a sputter-coating technique. The specimens were observed by field emission scanning electron microscopy. Electron imaging was performed at an accelerating voltage of 15 kV to investigate surface geometry with Zeiss EVO 40 scanning electron microscope at the magnification of 30,000x. 10 areas were randomly selected and analysed for each specimen (Figures 1(a) and 1(b)).

### 2.8. Statistical Analysis

The data was entered into MS Excel spreadsheet and analysed, using SPSS version 11 statistical software. Descriptive statistics were calculated for each variable for the three groups. Techniques applied were Student's *t*-test and one-way analysis of variance (ANOVA) followed by post hoc comparison by Bonferroni method. For each group and subgroup *p* < 0.05 was considered significant.

## 3. Results

### 3.1. Shear Bond Strength

Table 5 summarizes the mean values and standard deviations of SBS for all the tested zirconia and veneering ceramics, that is, Upcera, Ziecon, and Cercon. Among control groups (without artificial aging), Cercon showed the highest mean value (27.9 ± 6.54 MPa) followed by Ziecon (24.70 ± 6.76 MPa) and the lowest mean value was shown by Upcera (24.43 ± 7.13 MPa). The statistical difference was found to be nonsignificant among them (*p* = 0.66). Among the test groups (with artificial aging) Cercon showed the highest mean value (24.20 ± 8.87 MPa) followed by Upcera (24.0 ± 7.53 MPa) and the lowest mean value was shown by Ziecon (23.9 ± 8.03 MPa). The statistical difference was found to be nonsignificant among the subgroups (*p* = 0.99).

### 3.2. Fracture Modes

Table 6 presents the fracture analysis results in percentage. None of the test groups demonstrated adhesive failure. With VITA VM9 veneer, Ziecon and Cercon in control group demonstrated 90% cohesive failure. As for the zirconia of test group, Upcera and Cercon showed 30% combined failure.

### 3.3. Phase Transformation

Table 7 summarizes the mean of phase transformation (m-phase fraction) Vol% values and their respective standard deviation of all the three groups, that is, Upcera, Ziecon, and Cercon. Among control groups (without artificial aging), Cercon showed the highest mean value (12.6 ± 1.15%) followed by Ziecon (6.49 ± 0.77%) and the lowest mean value was shown by Upcera (6.08 ± 1.16%). The statistical difference was found to be significant among the subgroups (*p* = 0.00). Among the test groups (with artificial aging) Cercon showed the highest mean value (14.3 ± 0.70%) followed by Upcera (12.4 ± 1.9%) and the lowest mean value was shown by Ziecon (11.0 ± 0.18%). The statistical difference was found to be significant among the subgroups (*p* = 0.04).

### 3.4. Surface Grain Size

Table 8 summarizes the mean of surface grain size and their respective standard deviation of all the three groups, that is, Upcera, Ziecon, and Cercon. Among control groups (without artificial aging), Upcera showed the highest mean value (0.43 ± 0.039 *μ*m) followed by Ziecon (0.41 ± 0.064 *μ*m) and the lowest mean value was shown by Cercon (0.39 ± 0.060 *μ*m). The statistical difference was found to be nonsignificant among the subgroups (*p* = 0.532). Among the test groups (with artificial aging) Upcera showed the highest mean value (0.42 ± 0.034 *μ*m) followed by Ziecon (0.40 ± 0.05 *μ*m) and the lowest mean value was shown by Cercon (0.40 ± 0.032 *μ*m). The statistical difference was found to be nonsignificant among the subgroups (*p* = 0.781).

## 4. Discussion

Based on the results obtained, the proposed hypothesis was accepted. There is no significant difference in the mean values of shear bond strength among all samples for both aging and nonaging groups. Hence the proposed hypothesis was accepted and probably multicentric trials could be taken to get the explicable results. Zirconia is classified as a high strength ceramic material; it was introduced for use in dentistry as a biomaterial with unsurpassed mechanical properties. Its clinical application expanded from single crowns, short-span fixed partial denture, and multiunit full-arch zirconia frameworks to implant abutments and complex implant superstructures [13–15]. There is no doubt that if we compare the strength of zirconia framework in relation to shear bond strength between veneered ceramic and zirconia, the weakest link on comparative note is the bonding between zirconia and veneering zirconia. In the presence of water molecules the outer tetragonal grains of zirconia may transform into monoclinic grains [3]. This leads to a cascade of events as the transformation of one grain results in local volume expansion and causes stress to neighboring grains [16]. t~m transformation induced by aging in the humid environment of the oral cavity is commonly referred to as low-temperature degradation (LTD). At oral temperatures, the transformation proceeds very slowly [17].

### 4.1. Effect of Aging on Bond Strength

The results showed that there is no significant difference in the mean values of shear bond strength among all samples (Table 5). Similar results were obtained by studies done by Al-Dohan et al. (2004), Aboushelib et al. (2005), Ozkurt et al. (2010), and Aboushelib et al. (2004), wherein they obtained SBS values of zirconia veneered porcelain ceramic in the range of 27 MPa-28 MPa. However the results of our study are in disagreement with the low bond strength values (9.4 MPa–26 MPa) obtained by Guess et al. (2008). This difference in bond strength values could be attributed to the type of veneering ceramic used with the zirconia framework. In the present study between the aged and nonaged samples, aging did not make any clinically significant difference in the shear bond strength; that is, in clinical restorations the bond between the zirconia core and veneered ceramic would not be affected by time [18].

Another factor that may affect the shear bond strength is the multiple firing of veneer ceramic over zirconia core; due to multiple firing there is relaxation of residual stresses and phase changes from tetragonal to monoclinic transformation [19]. This not only affects the strength of the structure, but also may affect the core-veneer bond strength. Surface lifts that occur during tetragonal monolithic transformation can reduce the core-veneer bond strength [20].

### 4.2. Type of Bond Failure

Zirconia veneered samples of all groups (aged as well as nonaged) after SBS test showed combined and cohesive (within veneer) bond failure as seen under stereomicroscope, with the predominance of cohesive failure in the veneer layer with no adhesive failure (Table 6). Similar results were found in earlier studies by Studart et al. (2007), Guess et al. (2008), and Aboushelib (2006) who investigated the type of bond failure after SBS test. The failure mode observed for ceramic systems was mainly combined (adhesive at the interface and cohesive in the veneering ceramic) and rest of them were cohesive, that is, chip off in veneer material. In contrast, Ozkurt et al. (2010) in their study on type of bond failure in zirconia veneered specimens have found that, in all the test groups, both adhesive and combined failures occur between the zirconia cores and their veneering ceramics. The clinical implication of these findings is that in zirconia ceramic systems the interface of the bonded restorations came out to be stronger as there are more of chip-off fractures of the veneering ceramic and delamination rather than catastrophic failure of the core structure. The bond strength between zirconia and the veneering ceramic was higher than the cohesive strength of the veneering ceramic. In other words, the weakest link was not the interface but the veneering ceramic itself. Adhesive failure does not occur in the presence of a good bond between compatible ceramic core and veneering materials. Hence, to realize the benefit of the high strength of zirconia frameworks, the strength of veneering ceramics needs to be improved.

### 4.3. Phase Transformation

In the present study different analytical methods are applied, namely, XRD and SEM, to analyse the aging characteristics of the zirconia ceramics.

ISO Standards allow a maximum of 25% of m-phase to be present [21], post accelerated aging test conducted at 134°C with 2-bar pressure for 5 hrs for any commercial zirconia ceramics to be used in dentistry. XRD analysis results of our study among all the three zirconia samples, namely, Upcera, Ziecon, and Cercon, revealed that, after aging treatment, the m-phase content on the zirconia surfaces in all samples was found to be increased after aging relative to the tetragonal phase peak (Table 7). Cercon samples were observed to be most sensitive to the aging treatment and this was reflected as a large % increase of m-phase content on the surface of the sample (14.3%). This was followed by Upcera (11.4%), and Ziecon (11.0%) was observed to be least affected by aging treatment.

The present study on simulated aging has shown that LTD is observed to be insignificant over a simulated period of 15 years of clinical usage.

The literature is well supplemented with research on phase transformation and its effect on physical properties such as flexural strength and fracture toughness. However, very few studies have evaluated the quantitative phase transformation within zirconia frameworks due to LTD and the correlation of phase transformation with the bond strength between zirconia substructure and veneering ceramic.

In a study Xiao et al. observed the change in the monoclinic phase and compared the low-temperature resistance aging performances of the three clinical frequently used zirconia core materials Lava Frame, Cercon Smart, and Upcera before and after aging. XRD analysis showed that the m-phase contents of the three zirconia materials increased by prolonging the aging time, where in Upcera zirconia was the most sensitive to the aging treatment [22].

The differences in LTD observed in the three different brands of yttria-stabilized zirconia ceramics may be attributed to differences in grain sizes, distributions of the grains, and additives (e.g., binder for the pressing step) [23]. Other factors like technique of compacting the powdered zirconia into block may also influence the homogeneity and the density distribution of the material, hence the strength of the restorations. Difference in the sintering techniques (presintering and final sintering) may also cause the variation in material properties [24].

### 4.4. Grain Size

Control of grain size in zirconia ceramics is important to maximize the mechanical properties and minimize the possibility of LTD. Grain size may also affect the bond strength; a SEM analysis among zirconia groups of Upcera, Ziecon, and Cercon revealed that, after postaging treatment, the grain sizes in all the three zirconia ceramics were found to be insignificantly the same as that of samples without aging (Table 8). The results were in support of Kim et al. that the bond strength is not affected if the percentage of monoclinic content is restricted to up to 14% and a good bond between ceramic and veneering material was present at this % age of monoclinic content; also this % age of phase transformation showed no influence on the surface grain size in zirconia within all samples of the groups [25]. However zirconia has been shown to behave unpredictably in response to stress due to the phase transformation; it is very important to understand that the above results may be totally in reverse due to the effect of stress generated by clinical function such as masticatory forces, surface cracks, premature contact, or any other mechanism that can lead to stress formation within the prosthesis.

The strength and other physical properties of zirconia are related to the quality of the zirconia block, that is, its composition, method of compaction and converting into a blank, and homogeneity within the blank. Sintering mechanism may have a determinant influence on the phase transformation, grain size, and the physical and mechanical properties. A wide scope of research is open to investigate further the challenges in improving the properties of zirconia restorations. The study is in vitro, where clinical situations have been simulated. Therefore, the results of this study may not directly transpolate into serviceability of the prosthesis in clinical setting. However, the results do indicate the acceptable values of bond strength upon aging of all commercially available zirconia used in the study as compared to samples without any aging procedure.

## 5. Conclusion

From the present study it can be concluded that 15 yrs of clinical usage as induced by artificial aging has no effect on the bond strength of zirconia and the veneering ceramic. The effect of stress generated by clinical usage such as masticatory forces, surface cracks, premature contact, or any other mechanism leads to stress formation within prosthesis which could not be simulated in the study samples. This concentration of stress with the passage of time (aging) could affect the performance of zirconia based restorations clinically.

Within the limitations of the study, following conclusions were drawn:

(1) Bond strength between zirconia and veneering ceramic in all samples was comparatively same (24.43 MPa to 27.9 MPa) and aging of 15 years had no effect on bond strength (23.9 MPa to 24.20 MPa).
(2) The zirconia veneer interface came out to be stronger than the strength of the veneering ceramic; on average 70% of the samples have shown combined failure; hence to improve the longevity of zirconia restorations the strength of veneering ceramic has to be improved.
(3) Bonding of zirconia veneered restoration is not influenced if the phase transformation (% of monoclinic content) is up to 14%. ISO Standards allow a maximum of 25 wt% of m-phase to be present, post accelerated aging test conducted at 134°C with 2-bar pressure for 5 hrs for any commercial zirconia ceramics to be used in dentistry.
(4) Surface grain size (0.40 ± 0.032 *μ*m–0.42 ± 0.034 *μ*m) may not be affected by the phase transformation (% of monoclinic content) if the zirconia veneered restorations are placed in oral cavity for 15 years (as simulated by artificial aging).

## Figures and Tables

**Table 1:** Zirconia systems evaluated in this study.

Name	Composition	Manufacturer
(1) Cercon	ZrO_2_ (>93%), Y_2_O_3_ (4.95–5.35%), HfO_2_ (0.8%), Al_2_O_3_ (0.15–0.35%), trace elements (SiO_2_, NaO_2_, Fe_2_O_3_)	Cercon DeguDent, Germany
(2) Ziecon	ZrO_2_ (94–96%), Y_2_O_3_ (4.05–6.0%), HfO_2_ (1-2%), Al_2_O_3_ (0–0.1%), trace elements (SiO_2_, NaO_2_, Fe_2_O_3_)	Jyoti Ceramics, Nashik, India
(3) Upcera	ZrO_2_ + HfO_2_ + Y_2_O_3_ (99.6 wt%), Al_2_O_3_ (<0.5 wt%), SiO_2_, NaO_2_, Fe_2_O_3_ (<0.2%)	Upcera, Shenzhen, China

**Table 2:** Firing schedules of zirconia (as provided by the manufacturer).

Core material	Manufacturer	Sintering temperature	Sintering cycle time
Cercon	Cercon DeguDent, Germany	1,350°C	8 hrs
Ziecon	Jyoti Ceramics, Nashik, India	1500°C	8 hrs
Upcera	Upcera, Shenzhen, China	1,450°C–1500°C	8 hrs

**Table 3:** Properties of veneering material as provided by the manufacturer.

Veneering material	Manufacturer	Flexural strength (MPa)	CTE^**∗**^
VM9	VITA Zahnfabrik, Bad Säckingen, Germany	96	8.8–9.2

^*∗*^Coefficient of thermal expansion in 10^−6^/K between 25 and 500°C.

**Table 4:** Firing schedules of the veneering ceramic according to manufacturer.

Veneering ceramic	Temperature (°C)	Time (min)	Heating rate (°C/min)	Firing temperature (°C)	Holding time (min)
Liner					
VITA VM9	500	6	55	930	1

Dentin					
VITA VM9	500	6	55	910	1

**Table 5:** Comparison of shear bond strength (MPa) in all the groups showing mean, standard deviation, and significance for the samples within subgroups.

	Upcera	Ziecon	Cercon	*p* value (significance < 0.05)
Comparison in between subgroups without artificial aging				
Mean (*µ*m) ± SD	24.43 ± 7.13	24.70 ± 6.76	27.9 ± 6.54	0.66

Comparison in between subgroups with artificial aging				
Mean (*µ*m) ± SD	24.0 ± 7.53	23.9 ± 8.03	24.20 ± 8.87	0.99

**Table 6:** Type of bond failures after debonding of the samples among all the three groups as examined under stereomicroscope.

Groups	Subgroups Control = without artificial aging Test = with artificial aging	Number of samples showing cohesive failure	Number of samples showing combined failure
Upcera	Control (*n* = 10)	9 (*90% cohesive*)	1 (*10% combined*)
	Test (*n* = 20)	14 (*70% cohesive*)	6 (*30% combined*)

Ziecon	Control (*n* = 10)	8 (*80% cohesive*)	2 (*20% combined*)
	Test (*n* = 20)	15 (*75% cohesive*)	5 (*25% combined*)

Cercon	Control (*n* = 10)	8 (*80% cohesive*)	2 (*20% combined*)
	Test (*n* = 20)	14 (*70% cohesive*)	6 (*30% combined*)

**Table 7:** The comparison between the mean of phase transformation (m-phase fraction) Vol% values and their respective standard deviation of all the three groups, that is, Upcera, Ziecon, and Cercon, within each subgroup.

	Upcera	Ziecon	Cercon	*p* value (significance < 0.05)
Comparison in between subgroups without artificial aging				
Mean (%) ± SD	6.08 ± 1.16	6.49 ± 0.77	12.6 ± 1.15	0.00^*∗*^

Comparison in between subgroups with artificial aging				
Mean (%) ± SD	11.4 ± 1.9	11.0 ± 0.18	14.3 ± 0.70	0.04^*∗*^

^*∗*^Significant.

**Table 8:** Comparison of surface grain size (*µ*m) of zirconia in all the groups showing mean, standard deviation, and significance for the samples within subgroups.

	Upcera	Ziecon	Cercon	*p* value (significance < 0.05)
Comparison in between subgroups without artificial aging				
Mean (*µ*m) ± SD	0.43 ± 0.039	0.41 ± 0.064	0.39 ± 0.060	0.532^*∗∗*^

Comparison in between subgroups with artificial aging				
Mean (*µ*m) ± SD	0.42 ± 0.034	0.40 ± 0.05	0.40 ± 0.032	0.781^*∗∗*^

^*∗∗*^Non significant.
